# 

**Published:** 1932

**Authors:** 


### CONTENTS.

| | Page |
|---|---|
| Suprarenal Hypertrophy. By J. R. Charles, M.D., F.R.C.P. | 115 |
| Suprarenal Virilism. By J. Antony Birrell, M.D., M.R.C.P. | 119 |
| The Management of the Ear, Nose and Throat in Influenza. By A. J. Wright, M.B., F.R.C.S. | 123 |
| Notes on the Treatment of Some Common Ocular Affections. By E. R. Chambers, F.R.C.S., D.O.M.S. | 131 |
| Notes on Surgical Jaundice. By C. A. Moore, M.S., Ch.M., F.R.C.S. | 139 |
| Insomnia. By R. G. Gordon, M.D., D.Sc., F.R.C.P.E. | 147 |
| Reviews of Books | 157 |
| Editorial Notes | 166 |
| Meetings of Societies | 170 |
| The Medical Library of the University of Bristol | 172 |
| Local Medical Notes | 174 |

